# Convergence of External Crohn’s Disease Risk Factors on Intestinal Bacteria

**DOI:** 10.3389/fimmu.2015.00558

**Published:** 2015-11-03

**Authors:** Alexander Oberc, Brian K. Coombes

**Affiliations:** ^1^Department of Biochemistry and Biomedical Sciences, McMaster University, Hamilton, ON, Canada; ^2^Michael G. DeGroote Institute for Infectious Disease Research, Hamilton, ON, Canada; ^3^Farncombe Family Digestive Health Research Institute, Hamilton, ON, Canada

**Keywords:** Crohn’s disease, adherent-invasive *E. coli*, gastroenteritis, antibiotics, microbiota

## Abstract

Crohn’s disease (CD) is an immune-mediated intestinal illness that significantly compromises health in many developed countries. Although definitive causes remain elusive, the required contribution of microbes in the progression of disease has become an accepted concept. Known CD risk factors, such as antibiotic use and acute infectious gastroenteritis, may impact the gut. This concept is now being explored with a view toward understanding the beneficial and unfavorable microbes that may be altered in numbers during such external insults. A comprehensive understanding of the microbial component to CD could be useful clinically as future therapies may focus on preventing risk exposures on susceptible individuals, eliminating harmful microbes, or restoring a protective gut microbiome. Here, we examine how acute infectious gastroenteritis and antibiotic exposure may impact the gut microbiota in the context of inflammation in CD.

## Introduction

Crohn’s disease (CD) is part of the inflammatory bowel disease spectrum that can affect any portion of the gastrointestinal tract. CD is becoming increasingly common in many high-income nations, particularly among the pediatric population (1). Due to its high prevalence and chronic disease course, CD imposes significant directs costs on the health care system, along with a high attendant societal cost (2). Since the classification of CD in the 1950s, significant investments have been made in understanding the root causes of the disease, as well as in new treatments to induce remission that target mainly the aberrant inflammatory response underlying the disease course. Although genetics are known to play an important role in CD (3, 4), penetrance is low for any one genetic risk allele. Furthermore, the sharp rise in CD in many countries over the past 40 years indicates that genetics alone does not fully explain the etiology of disease, but rather that environmental exposures may be antecedent to disease initiation and progression in a genetically susceptible population (5). Understanding the nature and origins of such environmental risk factors is of paramount importance in curbing the rise of CD worldwide.

Clinical and animal studies have provided robust data indicating that intestinal microorganisms, both luminal and tissue-associated, play an important role in CD pathogenesis (5–9). For example, a pathogenic role for luminal bacteria is consistent with the beneficial effects of experimental fecal stream diversion following ileocolonic resection (10) and levels of epithelial-adherent bacteria have been found to correlate clinically with CD severity (11, 12). Environmental exposures, such as infectious agents or use of certain xenobiotics, can create intestinal vulnerabilities, rendering one more susceptible to the functional and microbial changes associated with CD. In this review, we briefly summarize the microbial composition changes that have been described in CD patients, and discuss two CD risk factors – acute infectious gastroenteritis and exposure to antimicrobial agents – that are known to induce pervasive changes to the microbial composition in the gut. The potential for dysbiosis-inducing external insults such as these to select for the expansion of pathobiont-like bacteria that affect the inflammatory tone of the gut is discussed.

## Microbial Dysbiosis in Crohn’s Disease

The advent of high-throughput sequencing has allowed researchers to investigate the gut microbiome in health and disease states, which was previously difficult to study due to the immense quantity and diversity of species present as well as the difficulty in getting broad representation of the intestinal community by culturing methods (13). Several high-throughput methods have been developed over the past decade that have allowed researchers to identify intestinal bacteria based on genetic signatures. One method involves using fluorescence *in situ* hybridization (FISH) probes designed to label certain groups of bacteria. These labeled bacteria can then be quantified using flow cytometry (FCM-FISH) (14). This method has the advantage of being relatively simple and inexpensive; however, the FCM-FISH method has relatively poor resolution and can only identify broad changes in bacterial groups. High-throughput sequencing has set a new benchmark, allowing researchers to analyze the population structure of complex microbial communities (15). In this regard, 16s rRNA is a useful genetic marker as it is universally present among all bacteria. It contains both variable regions useful for genomic classification and conserved regions that can be used to design universal primers. The choice of primers is extremely important, as even “universal” primers may poorly amplify some bacterial families. Although sequencing larger segments of the 16s rRNA region provides more resolution, most high-throughput sequencing technologies are limited to sequencing several hundred base pairs. For this reason, most studies analyze only one or several variable regions of the 16s rRNA gene. Nonetheless, even short reads of variable regions around 100 bases are usually sufficient to differentiate many microbes to the genus level, although using different variable regions can lead to different results (16). When comparing the results of 16s rRNA sequencing, it is important to consider differences in primer choice and the length of DNA sequenced from each fragment. Additionally, differences in data clean up as well as data analysis can lead to additional variability. For example, different analysis procedures between groups can result in considerable discrepancies in data generation and interpretation that should be taken into consideration when comparing observations from different publications. Nevertheless, some themes are emerging from the literature, which are discussed below.

The human microbiome is composed primarily of anaerobes within the bacterial phyla *Firmicutes* and *Bacteriodetes*. One of the first studies to investigate the CD microbiome examined fecal bacteria from six healthy individuals and six CD patients using both 16s rRNA sequencing and FCM-FISH (17). All CD patients were in remission, had no exposure to antibiotics for at least 3 months, and had previous ileal involvement in the disease course. In this study, CD patients had much lower microbial diversity than the control group, with 88 ribotypes represented in healthy subjects compared to only 54 in CD patients. This change was largely due to decreased diversity in the *Firmicutes* phylum, in particular, the *Clostridium leptum* group. *C. leptum* are known to produce butyrate, a short chain fatty acid (SCFA) and energy source for the intestinal epithelium. Butyrate has also been shown to down regulate pro-inflammatory mRNA within enterocytes and to acidify the intestinal lumen that is thought to inhibit certain pathogens such as *Salmonella* and *Escherichia coli* (18, 19). A similarly designed study using FCM-FISH analyzed fecal samples from 14 CD patients and 13 healthy controls that had not received sulfasalazine, antibiotics, or laxatives in the month prior to analysis (20). Using FISH with group-specific probes the study also found that *C. leptum* was significantly decreased in the CD group.

A larger study examined intestinal biopsy samples rather than fecal samples, from CD and UC patients and healthy controls (*n* = 190) using 16s rRNA sequencing (21). *Bacteroidetes* and *Lachnospiraceae* groups were greatly decreased in these subjects, whereas *Actinobacteria* and *Proteobacteria* groups were increased. Another study investigated both fecal and mucosal samples from healthy (*n* = 27), CD (*n* = 121), and UC patients (*n* = 75) utilizing 16s rRNA sequencing of V3–V5 regions (22). *Roseburia*, *Phascolarctobacterium*, and *Ruminococcaceae* were all reduced in CD patients. These microbial groups are also important producers of SCFA. In ileal CD patients, *Ruminococcaceae* and *Faecalibacterium* groups were particularly reduced while *Escherichia/Shigella* species were enriched, a finding that has been reproduced in several studies. Interestingly, the use of 5-ASA was strongly correlated with a decrease in *Escherichia/Shigella*. Various genera within *Clostriales* order were decreased in patients treated with antibiotics, possibly due to their sensitivity to ciprofloxacin/metronidazole often used in CD treatment. The genes involved in several major metabolic pathways have been well characterized in model bacteria. Since many of the genome of gut microbes have been sequenced, it is possible to predict if a microbe lacks a particular classical pathway based on an absence of conservation to known genes typically involved in that pathway. Overall, microbes from IBD patients had decreased pathways for amino acid synthesis coupled with increased amino acid uptake pathways. There were also increased levels of carbohydrate and lipid uptake and metabolism pathways, particularly in ileal CD. Many of these changes may aid in the metabolism of mucin, which is often overproduced during intestinal inflammation. The reduction in amino acid synthesis resembles phenotypes elicited by murine pathobionts known as segmented filamentous bacteria (23, 24). The microbiome showed increased glutathione uptake, which may be important for surviving oxidative stress from inflammation (25). Finally, the ileal CD metagenome was enriched in bacterial secretion systems, in particular, the type II secretion system that is often used in the export of toxins (26).

Another recent study analyzed the treatment-naïve microbiome of pediatric CD patients (*n* = 447) and healthy controls (*n* = 221) using tissue biopsies and feces and sequencing the 16s rRNA V4 region (27). When comparing intestinal biopsy samples from healthy individuals to CD patients, the *Bacteroidales* and several species within the *C. leptum* groups had lower abundance in pediatric CD patients in accordance with previous studies. Several bacterial families that were found to be more abundant were *Enterobacteriaceae*, *Pasteurellaceae*, *Veillonellaceae*, *Neisseriaceae*, and *Fusobacteriaceae*. The use of antibiotics in patients already diagnosed with CD is controversial (28, 29). However, in this study, a subset of CD patients had been treated with antibiotics providing for a separate analysis. When this group was analyzed separately, it was found that antibiotic exposure amplified the microbial dysbiosis associated with CD. Importantly, in new onset patients, the microbial dysbiosis was poorly reflected in the stool compared to tissue biopsy samples, which might suggest that the bacterial community of pathogenic significance in the disease is tissue associated.

**Figure 1:**
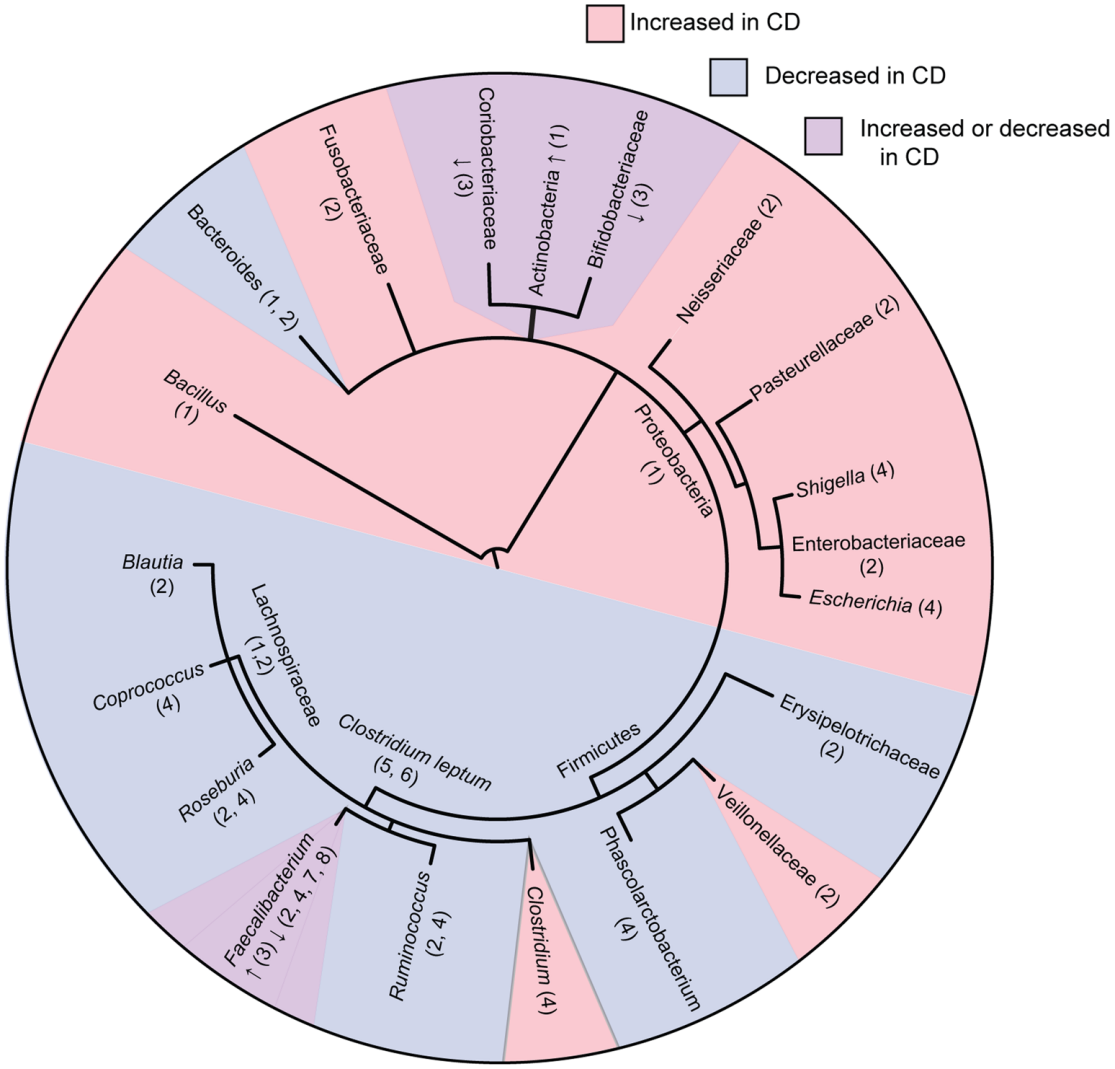
**Phylogenetic tree of bacterial groups associated with increased or decreased abundance in the gut microbiome of Crohn’s disease patients**. In cases were bacterial groups have been associated both increases and decreases, studies showing increased abundance are indicated with “↑” and studies showing decreased abundance are indicated with “↓.” Numbers indicate references as follows; 1, Frank et al. (21); 2, Gevers et al. (27); 3, Hansen et al. (31); 4, Morgan et al. (22); 5, Sokol et al. (20); 6, Manichanh et al. (17); 7, Sokol et al. (32); 8, Sokol et al. (33). Tree topology was created using phyloT (http://phylot.biobyte.de/) and the figure was made using ITOL (http://itol.embl.de/).

Overall, these studies consistently show that CD is associated with decreased abundance and diversity of the *Firmicutes* phylum with an increased abundance in the *Proteobacteria* phylum (Figure 1). Tissue samples appear to be more indicative of dysbiosis compared to fecal samples at least in early disease. Although *Firmicutes* appear decreased in overall abundance in CD, some specific members of the *Firmicutes* are conversely increased. These studies occasionally differ on the particular groups of bacteria that are elevated or reduced in CD patients. These differences may be due to differences in sample type (luminal vs. tissue) as well as differences in patient populations (patients with active disease vs. remission, treated patients vs. treatment naïve), or in method of analysis. How these shifts in microbial ecology contribute to the pathophysiology of CD remains an active area of research. A recent study using a murine model of CD demonstrated that bacteria gut dysbiosis played a causal role in intestinal inflammation (30). Several mechanisms may explain why dysbiosis is associated with CD. It is possible that dysbiosis contributes to CD because the loss of SCFA producing bacteria has multiple impacts on the gut environment, including impaired survival of enterocytes, increased production of inflammatory cytokines, and decreased suppression of potentially pathogenic *Proteobacteria*. It is also possible that dysbiosis is caused by the intestinal inflammation in CD. Bacterial species from *Proteobacteria* are facultative anaerobes that tend to have a higher resistance to reactive-oxygen species produced during inflammation, possibly giving them a selective advantage over the predominantly obligate anaerobes from *Firmicutes* and *Bacteroidetes*. These mechanisms are not mutually exclusive, and it is likely that microbial dysbiosis both contributes to and results from the intestinal inflammation seen in CD.

One member of the *Firmicutes* that has received much attention in the context of CD is *Faecalibacterium prausnitzii*, a major member of the *C. leptum* group of *Firmicutes* that is decreased in CD patients (32). In particular, a higher abundance of *F. prausnitzii* was associated with a higher rate of remission following resection surgery (33). *In vitro* analysis showed that this bacterium was capable of inhibiting NFκB and the secretion of pro-inflammatory cytokines, as well as stimulating the release of anti-inflammatory cytokines, such as IL-10. *In vivo* studies using dinitrobenzene sulfonic acid (DNBS) (34) and trinitrobenzenesulfonic acid (TNBS) (33)-induced colitis models showed that *F. prausnitzii* and its culture supernatant were capable of attenuating inflammation when introduced both orally and into the peritoneal cavity. An intriguing recent study has identified a protein secreted by *F. prausnitzii* that may explain its anti-inflammatory properties (35). However, the exact role of *F. prausnitzii* in the pathogenesis of CD has yet to be elucidated. Two studies of pediatric patients with newly diagnosed, untreated CD conflictingly showed increased (31) and decreased (27) levels of in *F. prausnitzii* compared to healthy controls. It is possible that the decreased levels of *F. prausnitzii* are a characteristic of adult CD but not pediatric CD. It is also possible that *F. prausnitzii* is lost due to medications used to treat CD such as antibiotics rather than having a direct link to disease expression. Additional research is needed to determine how levels of *F. prausnitzii* might dictate the fine balance between health and disease in the gut.

## Infectious Gastroenteritis as a Risk Factor for CD

Amply powered clinical studies show that acute infectious gastroenteritis caused by enteric pathogens (including *Salmonella* and pathogenic *E. coli*) increases the short- and long-term risk of developing CD (36–38) (Table 1). In one study, Garcia-Rodríguez et al. compared a cohort of individuals with acute gastroenteritis (*N* = 43,013) with matched controls without documented gastroenteritis (*N* = 50,000), excluding individuals with previous IBD diagnosis (36). Over a mean follow-up period of 3.5 years, there was an increased risk of developing IBD after a single episode of acute gastroenteritis (HR 2.4 [1.7–3.3]). The association was stronger with CD compared to ulcerative colitis (UC), particularly in the first year after gastroenteritis (HR 6.6 [1.9–22.4]). A second case–control study by Porter et al. compared individuals diagnosed with CD with healthy controls (37). The study examined previous diagnoses of infectious gastroenteritis in both groups. Patients with IBD were more likely to have had a previous diagnosis of infectious gastroenteritis (AOR 1.40 [1.19–1.66]). Once again, the risk was higher for CD (AOR 1.54 [1.17–2.04]) than for UC (AOR 1.36 [1.08–1.72]). Finally, a retrospective study compared cohorts of healthy individuals in Denmark to those diagnosed with acute gastroenteritis caused by either *Salmonella* or *Campylobacter*, tracking subsequent IBD diagnoses over the following 15 years (38). Overall, the gastroenteritis group had an increased risk of subsequent IBD diagnosis over the entire 15-year period (HR 2.9 [2.2–3.9]), with the greatest risk occurring in the first year following an episode of acute gastroenteritis.

**Table 1:** **Epidemiological studies associating infectious gastroenteritis and Crohn’s disease**.

Study	Type	Location	Population size	Type of association	Risk [95% CI]
Garcia Rodriguez et al. (36)	Retrospective cohort	United Kingdom	IGE: 43,013 Cont: 50,000	Overall	HR 6.6 [1.9, 22.4]
Porter et al. (37)	Case–control	United States	CD: 1,037 Cont: 11,646	Overall	OR 1.54 [1.1, 2.04]
Gradel et al. (38)	Case–control	Denmark	IGE: 13,148 Cont: 26,216	Overall	HR 3.0 [1.7, 5.3]

*IGE, infectious gastroenteritis; HR, hazard ratio; OR, odds ratio*.

Due to the observational nature of these studies, it is difficult to determine whether infection predisposes individuals to CD or if individuals with a susceptibility CD are more susceptible to infection. It is possible that low-grade inflammation in prodromal CD may lower the colonization resistance to enteric pathogens, a phenomenon has been demonstrated in murine models (39). Several studies have shown that infectious gastroenteritis is associated with microbial dysbiosis, albeit transiently (40, 41), that might underlie the microbial triggers of chronic disease. Though the mechanisms are currently unknown, the long-term risk of developing CD following acute infectious gastroenteritis might suggest that resident gut microbes could perpetuate inflammatory reactions in the post-infectious period. One central hypothesis is that exposure to infectious pathogens creates an environment favorable to colonization by other pro-inflammatory bacteria. These may be existing members of the microbiota typically restricted by microbial and host processes, or they may be acquired *de novo* through vulnerabilities created by the disruption of the resident microbiota during gastroenteritis. For example, human (42) and animal studies (43, 44) indicate that inflammation during gastroenteritis selectively disrupts the resident intestinal microbiota in favor of *Enterobacteriacea*, such as *E. coli*. Whether such disruptions have long-term consequences on the host in the post-infectious period has not been rigorously studied.

## Antibiotics as a Risk Factor for CD

Antibiotics are commonly prescribed for a wide range of illness and are occasionally used in the treatment of CD (45). Antibiotics have long been known to have negative impacts on commensal microbes and are known to be an important risk factor for gastrointestinal illnesses, such as *Clostridium difficile* infection (46, 47). Several epidemiological studies have linked exposure to antibiotics with CD, which are summarized in Table 2. A case–control study in Canada compared antibiotic use in individuals with incident IBD diagnosis and non-IBD controls and found that patients diagnosed with IBD were more likely to have been prescribed antibiotics in the preceding 5 years before diagnosis (48). Of interest, there was a dose-dependent relationship between the number of antibiotics dispensed and the risk of subsequent IBD diagnosis. The risk of subsequent IBD diagnosis was highest 2 years after antibiotics and diminished over time. Nearly all antibiotics included in this study, with the exception of clindamycin, were associated with an increased risk of IBD diagnosis. Interestingly, metronidazole had the highest risk (AOR 2.86 [2.24–3.65]) followed by quinolones (AOR 1.45 [1.27–1.64]). Both of these antibiotics are also used as treatments for CD.

**Table 2:** **Epidemiological studies associating antibiotic treatment with subsequent CD diagnosis**.

Study	Type	Location	Population size (CD:control or cohort size)	Type of association	Risk [95% CI]
Card et al. (49)	Adult	United Kingdom	587:1460	2–5 years before CD diagnosis	OR 1.32 [1.05, 1.65]
	Case–control				
Margolis et al. (50)	Adult	United Kingdom	94,487	Overall	HR 1.62 [1.04, 2.53]
	Retrospective cohort				
Shaw et al. (48)	Adult	Canada	1025:22,346	2–5 years before CD diagnosis	AOR 1.29 [1.18, 1.40]
	Case–control				
Hildebrand et al. (53)	Pediatric	Sweden	1098:6550	Pneumonia <5 years old	OR 3.56 [1.79, 7.08]
	Case–control				
Hviid et al. (51)	Pediatric	Denmark	577:627	Overall	RR 3.41 [1.45, 8.02]
	Retrospective cohort				
Virta et al. (52)	Pediatric	Finland	233:2380	2 years before CD diagnosis	AOR 1.87 [1.37, 2.56]
	Case–control				

*OR, odds ratio; HR, hazard ratio; AOR, adjusted odds ratio; RR, relative risk*.

A case–control study using data from the UK examined patient medical history at least 5 years prior to diagnosis of CD (49). Again, patients who were later diagnosed with CD were more likely to have taken antibiotics (OR 1.32 [1.05–1.65]). When individual antibiotics were examined, only metronidazole and tinidazole (OR 1.71 [1.05–2.76]) and tetracylines (OR 1.33 [1.01–1.77]) were associated with subsequent CD. This study did not find a dose-dependent relationship between the numbers of antibiotics dispensed and overall CD risk.

A cohort study from the UK examined the association between oral tetracycline for the treatment acne in adults and adolescents and subsequent IBD diagnosis (50). Once again, there was an association between tetracycline use and IBD (HR 1.39 [1.02–1.90]) and a stronger association with CD in particular (HR 1.62 [1.04–2.53]). Furthermore, the risk of CD varied based on the type of antibiotic, with minocycline conferring the lowest risk (HR 1.28 [0.72–2.29]) followed by tetracycline (HR 1.61 [1.00–2.63]) and finally doxycycline (HR 2.25 [1.27–4.00]).

Several studies in pediatric patients have shown similar results. A study in Denmark followed all singleton children born between 1995 and 2003 (51) and tracked antibiotic prescriptions and IBD diagnoses. CD, but not UC, was associated with previous antibiotic prescriptions (RR 3.41 [1.45–8.0]). This relationship was dose dependent, with the highest risk being among children prescribed ≥7 antibiotics (RR 7.32 [2.14–24.9]). Interestingly, the risk of diagnosis was highest within 3 months of antibiotic exposure (RR 4.43 [1.88–10.44]). Penicillin V and extended spectrum penicillins conferred the highest risk (RR 2.92 [1.22–6.97] and RR 3.13 [1.33–7.40], respectively). Another study from Finland similarly found an association between antibiotic use 2 years preceding CD (AOR 1.87 [1.37–2.56]), but not UC, diagnosis (52). Cephalosporin antibiotics were associated with the highest risk. Finally, a Swedish study which used childhood infections as a proxy for antibiotic treatment found a position association between pneumonia, diagnosed at <5 years old, and subsequent CD (OR 3.56 [1.79–7.08]) (53).

These studies have provided evidence for a correlation between antibiotic use and subsequent diagnosis of CD; however, most of the studies do not agree on a specific class of antibiotic that confers the highest risk, which may relate to differences in patient populations or prescribing practices. Due to the observational nature of these studies, it is difficult to show causality between antibiotics and CD and it remains possible that the phenotype that makes one susceptible to CD may also make one susceptible to infections requiring antibiotics. Conversely, among patients already diagnosed CD, the use of antibiotics is associated with a reduced chance of flaring (54). Relatively short courses of antibiotics are known to lead to dramatic shifts and loss of diversity on the gut microbiome, which can persist for years (55). It is possible that antibiotic exposure may trigger an unfavorable microbial community structure that increases the potential for long-term dysbiosis and risk of CD in susceptible individuals. Microbiome comparison between CD patients with and without exposure to antibiotics supports this claim, showing that antibiotics magnify the dysbiosis signature associated with CD (27). Nonetheless, robust experimental evidence is needed to clarify the impact of antibiotic use on host susceptibility to disease.

## Tissue-Associated Bacteria are Increased in Crohn’s Disease

Inflammation in CD can involve both the small and large bowel and is accompanied by changes in the microbial composition and distribution at these sites (6, 13). Clinical observations are consistent in finding increased numbers of bacteria associated with the intestinal epithelium in Crohn’s patients (5, 12, 22). The intestinal mucosa, made up of the epithelium and underlying lamina propria, is a site rich in immune cell populations that protect the integrity of the epithelial surface and direct the innate and adaptive immune responses. Since this site is consistently breached in CD, attention has focused on the relationship between the innate immune system and the microbiota (56), with a particular emphasis on the bacteria that penetrate into this normally aseptic site. Examination of the mucosa in ileal CD has been informative. It is here that the density of *E. coli*, enriched in virulence and secretion pathways, is elevated as determined by culture and molecular methods (11, 22, 57). Use of bowel-specific aminosalicylates is associated with normalization of this *E. coli* bloom in ileal disease (22, 58), suggesting that inflammation might somehow benefit certain bacteria that are associated with the mucosal surface. This finding is important clinically because the severity of ileal disease has been shown to correlate directly with the density of mucosa-associated *E. coli* (12). The implication of course is that reducing the burden of mucosa-associated bacteria would have favorable outcomes on the disease course, possibly opening up new therapeutic avenues beyond immunosuppression. Together, these data support the general view that mucosa-associated *E. coli* are of pathogenic significance in the disease and that their abundance can be manipulated using pharmacologic treatments directed at host inflammation. These results stimulate many important and interesting questions. For example, they imply that the selective environment favoring *E. coli* expansion at the mucosal surface is inflammatory in nature. Although the exact source of this selection is not yet known, fruitful lines of investigation can be envisaged to uncover it. Furthermore, does the inflamed mucosa render a host more susceptible to *de novo* colonization by bacteria with pathobiont-like features, or is this expansion seeded from a resident population of opportunistic bacteria that exploit this favorable niche? Lastly, the genetically encoded bacterial adaptations needed for mucosal expansion in the inflamed gut are not known, but uncovering them would offer valuable insight into the evolutionary process that selects for such pathobionts. The upshot of this information would be new useful genetic markers for detecting pathobionts in hosts and other potential environmental reservoirs, and in identifying new potential targets for antimicrobial drug discovery.

## Adherent-Invasive *E. coli* in Crohn’s Disease

Much experimental and observational data implicate infectious agents in the initiation and maintenance of chronic inflammation in the intestine (6). *E. coli* is a Gram-negative species in the intestine where it can have a positive effect on gut homeostasis. However, through acquisition of virulence factors, such as toxins, adhesins, and secretion systems, *E. coli* can develop pathogenic traits that participate in intestinal and extraintestinal disease processes (59). Work originating from the laboratory of Dr. Darfeuille-Michaud identified *E. coli* in ileal biopsies from patients with CD (57), which they called adherent-invasive *E. coli* (AIEC) to reflect their atypical ability to adhere to mucosal epithelial cells and invade and survive within human cells. These *E. coli* were found to lack known virulence factors typical of pathogenic *E. coli*, including type III secretion systems or phage-encoded toxins, suggesting that they were a newly described pathovar. A series of papers followed that detailed the intracellular lifestyle of AIEC and the inflammatory response by cultured cells to AIEC infection (60, 61). AIEC are capable of replicating within macrophages and causing these cells to release high amounts of TNFα *in vitro* (62). It also appears possible that AIEC exploit cell phenotypes associated with genetic CD risk factors, such as defects in autophagy (63).

Several studies have now confirmed that AIEC are enriched in humans with CD, where they are six-times more likely to be isolated from ileal and colonic samples compared to healthy controls and represent the dominant bacterial species present (57, 64, 65). A growing body of work has uncovered genetic and phenotypic diversity among AIEC isolated from adults (12, 65–67), children (68, 69), and companion animals (70) indicating that different host environments can select for the AIEC phenotype. Since AIEC can be isolated from seemingly healthy individuals (albeit much less frequently than in Crohn’s), this genetic diversity suggests that interactions between AIEC and other CD risk factors might be needed to elicit disease in a subset of individuals.

In 2013, Small et al. established a novel model for chronic AIEC infection in conventional mice (8), that over time, develop transmural inflammation and fibrosis in the small and large intestine, the very hallmarks of CD. Importantly, this happens over timescales consistent with a progressive chronic disease without the need for foreign chemicals to induce inflammation. Using this model, it was shown that AIEC is resistant to innate antimicrobial defenses at the epithelial surface, including resistance to antimicrobial peptides released by Paneth cells and colonocytes (71). The molecular mechanism for this resistance involves, in part, the expression of a surface-localized protease that cleaves cationic host defense peptides to render them inactive. In addition to resistance to host-derived antimicrobial molecules, AIEC tend to be resistant to multiple xenobiotic antimicrobials, particularly in isolates obtained from patients with ileal disease (72). This likely reflects the selective environment in which AIEC evolve within hosts, as CD patients are often given antibiotics as part of their treatment regimen. As of yet, no studies have examined whether eradicating AIEC is a viable or efficacious strategy in the treatment of CD; however, animal models permitting chronic colonization and disease progression will help facilitate such studies in the future.

## Conclusion

Intestinal bacteria play an important role in the pathogenesis of CD in a manner that is incompletely understood. External risk factors, such as acute infectious gastroenteritis and antibiotic exposure, are moderately associated with CD, yet neither can be considered sufficient to elicit disease on their own. It is likely that a combination of genetic and environmental risk factors is necessary in the pathogenesis of CD; however, exactly how such a constellation of risk factors manifest in disease expression has been a challenging problem to address. The study of mucosal-associated *E. coli* with pathobiont-like properties has yielded promising new leads into the pathogenesis of CD but more work needs to be done to understand where such microbes come from, where they evolve their pathogenic features, and what the effect of their eradication might have on susceptible hosts. A mechanistic understanding of the pathogenic microbes in CD will offer more therapeutic targets and perhaps paradigm shifting approaches toward curative treatments for this disease.

## Author Contributions

AO and BC wrote the paper.

## Conflict of Interest Statement

The authors declare that the research was conducted in the absence of any commercial or financial relationships that could be construed as a potential conflict of interest.
